# Artificial Attractants: Implications for Disease Management in Deer

**DOI:** 10.1002/ece3.71013

**Published:** 2025-02-20

**Authors:** Kelsey Gritter, Margo Pybus, Mark A. Lewis, Evelyn Merrill

**Affiliations:** ^1^ Department of Biological Sciences University of Alberta Edmonton Alberta Canada; ^2^ Fish and Wildlife Stewardship Government of Alberta Edmonton Alberta Canada; ^3^ Department of Mathematics and Statistics University of Victoria Victoria British Columbia Canada; ^4^ Department of Biology University of Victoria Victoria British Columbia Canada

**Keywords:** attractants, chronic wasting disease, contacts, individual‐based model, mule deer, Odocoileus, simulation

## Abstract

Chronic wasting disease (CWD) is a prion disease that infects cervid species by direct and environmental transmission and is invariably fatal. CWD spread can be promoted by the attraction of animals to “hotspots” such as hay bales and grain bags stored in fields and at farm sites. The density and location of hotspots may impact contact rates. We used an individual‐based movement model of mule deer ( *Odocoileus hemionus* ) to investigate the effects of density and configuration of hotspots (hereafter artificial attractants, AA) on contact rates at a constant density of 1 deer/km^2^ during winter. The model tracks when two deer from the same or different groups come into contact under 6 AA densities (0–1 AA/km^2^) and 6 AA configurations. We compared placing AA randomly versus clustered around farms, and removing them randomly versus biased by proximity to preferred habitat. Overall, the number of unique contacts per individual and the number of unique deer visiting an AA increased, and the number of AAs used by each deer decreased as AA density declined. Selectively removing field attractants near preferred habitat resulted in a larger increase in contacts per deer, with deer contacting more and different individuals, fewer deer using the remaining AA, and fewer visits per AA than random removal. There was a greater increase in contact rates when reducing AA density at farms by randomly removing all AA at a farm compared to randomly removing individual AA across farms. Deer responses to AA removal may not be as straightforward as originally believed. Deer contacts may increase, not decrease, with AA removal because deer are attracted to the remaining AA. Under moderate deer densities, AA removal may require a broad‐scale, “all or nothing” approach to prevent deer from concentrating at remaining AA, but concomitantly lowering deer density needs further assessment.

## Graphical abstract

We used an individual‐based movement model of mule deer based on empirical data to investigate the effects of density and configuration of artificial attractants on contact rates during winter. Deer responses to artificial‐attractant removal may not be as straightforward as originally believed. Deer contacts may increase, not decrease, with artificial‐attractant removal because deer are attracted to the remaining artificial attractants.

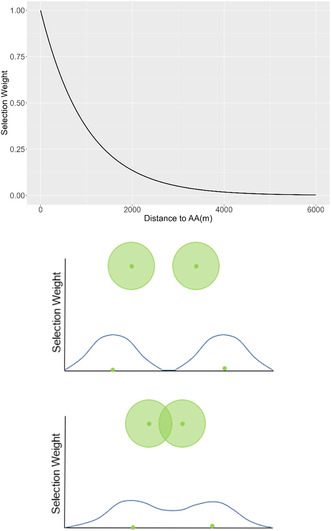

## 1 Introduction

Anthropogenic activities on landscapes can create natural and artificial hotspots of wildlife activity that can contribute to the spread of diseases. Supplemental feeding, baiting, and unintentional attractants, such as hay bales and grain bags, are common attractants for ungulates in North America (Sorensen 2014; Milner et al. 2014). Supplemental feeding sites are designed to attract large herbivores for viewing or hunting, to reduce herbivory or vehicle collisions on roads, or to influence population dynamics by supplying additional food resources (Sorensen 2014). Unintentional feeding includes depredation on agricultural crops, such as grain and canola, when they are growing or in storage units in the field of origin or in storage bins clustered around farms (Jerina 2012; Andreassen et al. 2005; Felton et al. 2017). Economic losses to farmers from depredation on stored crops and hay totaled nearly a quarter‐million dollars in damage in 1989 in the United States, with costs as high as hundreds of dollars per hectare (Austin et al. 1998; Menichetti et al. 2019; Wywialowski 1994). Most jurisdictions pay some level of compensation to farmers for crop damage or provide materials to fence out ungulates to prevent depredation on stored crops (Gooding and Brook 2014; Menichetti et al. 2019), but these programs depend on farmers following acceptable storage practices, such as erecting fencing and using repellents (Lemieux et al. 2000; Mysterud et al. 2021; Mysterud and Rolandsen 2019). Nevertheless, the aggregation of ungulates in winter due to improperly stored grains or accidental spillage remains a key problem in most agricultural areas (Mysterud et al. 2021; Sorensen et al. 2015).

In addition to the economic losses, aggregation of ungulates in winter at stored crops can lead to higher disease transmission (Cotterill et al. 2018). High use of artificial attractants (AA), such as grain bins, stored hay bales, and grain bags, facilitates direct transmission by increasing interactions among individuals at a site or indirect transmission where diseases are transmitted environmentally (Escobar et al. 2020; Sorensen 2014; Thompson et al. 2008). For example, individual deer have been shown to stay at AA 1.36–1.69 times longer as compared to natural feeding areas, with an increased number of contacts occurring among individuals from different groups (Cross et al. 2013; Sorensen 2014; Thompson et al. 2008). The layout of the feed also influences the amount of time white‐tailed deer spend at sites, with the most time spent where grain was spread out, followed by sites where the grain was in a pile, both of which were higher than at natural areas (Miller et al. 2003; Thompson et al. 2008). Infected animals also can contact individuals along travel routes to and from AA and, if infective agents are dropped or excreted along the routes, can spread a disease within the nearby home ranges of other individuals (Benavides et al. 2012). As a result, the role that AA play in disease transmission in an area will depend on the extent to which ungulates use AA, which in turn is contingent on the number of AA available, their spatial distribution relative to deer movement, and the availability of other natural, high‐quality forages and preferred habitat (Miller et al. 2003; White et al. 2018).

One disease of cervids where AA may be influencing the transmission and spread of the disease is chronic wasting disease (CWD, Sorensen 2014; Western Association of Fish and Wildlife Agencies (WAFWA) 2018). CWD is a 100% fatal prion disease in cervids where transmission occurs both directly through contact between individuals and indirectly through the environment (Williams and Miller 2003). CWD currently is found in wild cervid populations in 4 Canadian provinces and 35 states (U.S. Geological Survey 2024). In Alberta, CWD was first detected in wild deer in 2005 (Smolko et al. 2021). Alberta has maintained a hunter‐harvest surveillance program since before the first detection of CWD along the Alberta/Saskatchewan border. Surveillance data indicate that CWD is spreading from east to west, and province‐wide prevalence in 2023 was 30.5% in mule deer ( *Odocoileus hemionus* ) and 7.9% in white‐tailed deer ( *O. virginianus* ). Because there is no vaccine for CWD, the most common management approach for addressing CWD has focused on manipulating harvest strategies (Conner et al. 2021; Rivera et al. 2019). However, if AA play a significant role in CWD transmission (Western Association of Fish and Wildlife Agencies 2018), particularly in agricultural areas, additional research is needed to provide guidelines for best practices in reducing disease transmission at AA (Heberlein 2004; Peterson et al. 2002).

Given the logistical and social difficulties of eliminating AA in real landscapes, we use an individual‐based model (IBM, Gritter et al. 2024) as a first step to assessing the influence of AA density, configuration, and proximity to preferred habitat on contact rates to gain insight on their management for preventing disease transmission. We simulate deer contact rates, using Netlogo, in a landscape representative of central‐eastern Alberta, the area where CWD was first detected and currently is enzootic (Wilensky 1999). In this area, unintentional winter feeding sites associated with hay bales, grain bags, and grain bins are common (Ewald, pers. comm.; Government of Alberta 2021; Mejía‐Salazar et al. 2018). We hypothesized that at moderately high densities of AA, individual deer would have the highest contact rates with other deer because they are attracted to multiple AA within their attraction range, whereas as AA are removed and become more dispersed or AA density increases, contact rates would decrease because deer use primarily local, nearby AA, thereby sharing fewer sites and contacting fewer other individuals (e.g., Becker et al. 2018; Sah et al. 2018). This may be contingent on the configuration of AA or their proximity to preferred habitat, such as woody cover, to which deer are strongly attracted in the prairie‐parklands of Alberta (Habib et al. 2011; Nobert et al. 2016). Understanding if thresholds in contact rates might emerge will be key to guiding future regulations for how to effectively manage AA to minimize transmission and spread of CWD and other diseases.

## 2 Methods

### 2.1 Study Area

**FIGURE 1:**
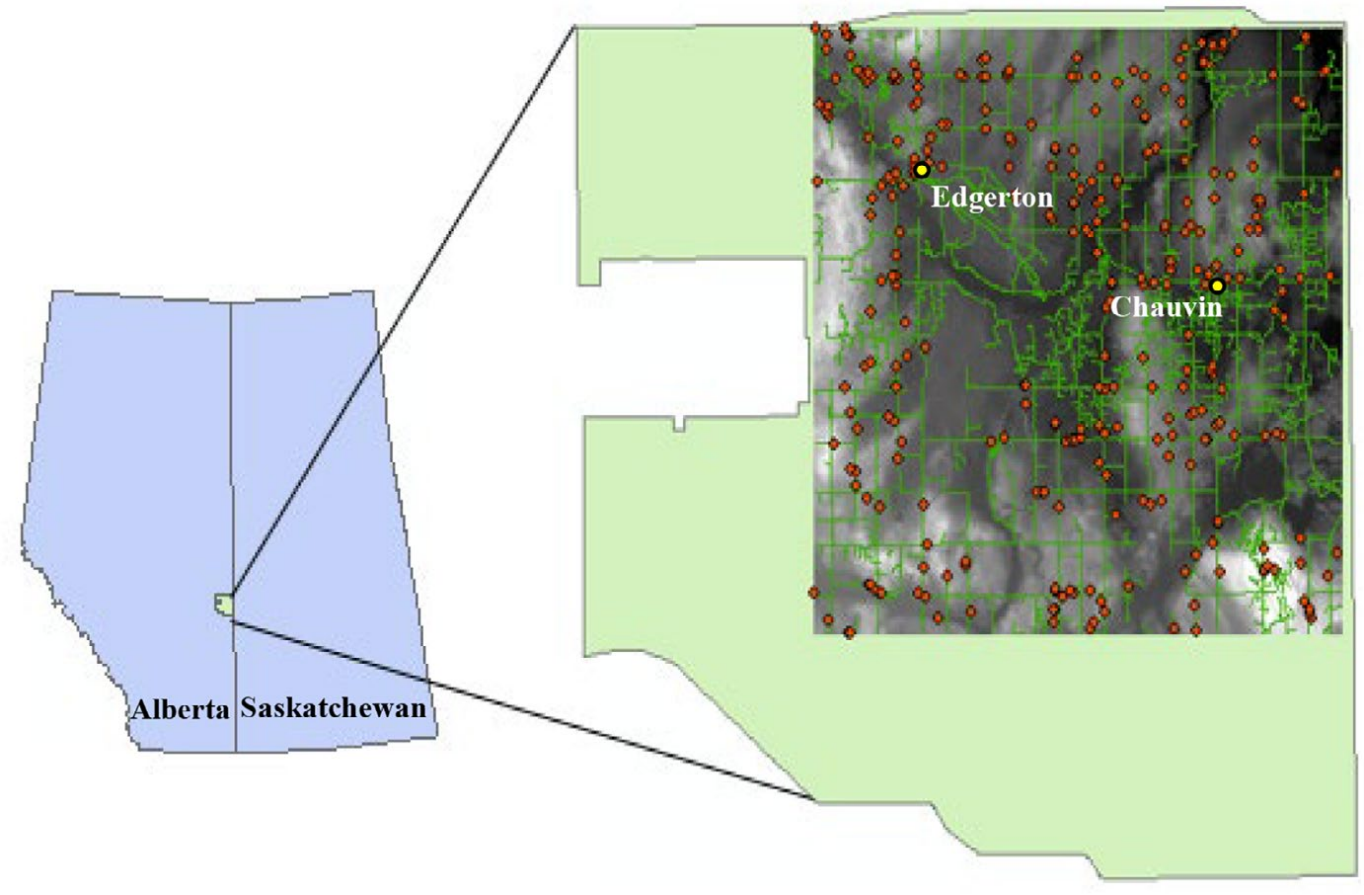
Location of simulation area in eastern Alberta, Canada showing wildlife management unit 234 in green. Simulation area (gray shaded) displays elevation, roads in green, and farm sites in red (Altalis 2018, 2020; The Municipal District of Provost No. 52 2019; The Municipal District of Wainwright No. 61 2020).

The landscape is a 1440‐km^2^ area in Wildlife Management Unit (WMU) 234 in eastern Alberta, in the CWD disease zone (Figure 1). The area is rolling hills ranging in elevation from 553 to 782 m within the parkland ecosystem (Meijer and Karpuk 1999). The landscape is heterogeneous with a mix of agricultural cropland (48%), grassland and pastures (18.9%), and woody cover (24%) consisting of deciduous tree stands (*Populus* spp., 20.1%) and tall shrubland ( *Elaeagnus commutata* , *Salix* spp., *Prunus* spp., and *Amelancier alnifolia*, 3.9%). Agricultural crops mainly consist of canola (*Brassica* spp.), wheat (*Triticum spp*.) and alfalfa (*Medicago* spp.) or perennial crops (Dobbin et al. 2023). The area has multiple small creeks and streams. Land use is primarily agricultural and cattle farming, with oil and gas extraction throughout the area. Winter temperatures ranged between ~−30° and ~15°C (Lloydminster and Edgerton weather data) and monthly snow depth generally ranged from 0 to ~45 cm.

### 2.2 Deer Movement Model and Attraction to AA

We used a movement model to move mule deer groups across the landscape based on sex‐specific integrated step‐selection functions (iSSF) derived from GPS‐collared mule deer with movement being biased toward a home‐range centroid and influenced by social grouping. Groups did not change size across the winter with a mean size of 6.6 and sex ratio of 70:30 females to males, corresponding to population‐level data for this area (unpublished data). At the beginning of each simulation, the centroid of a group of deer was located across the landscape according to the highest predicted values from an empirically based home‐range resource selection function (RSF). Individual deer within groups were moved in 2‐h timesteps responding to landscape covariates at a 30 by 30‐m scale, and a Von Mises distribution of turn angles produced home‐range behavior by incorporating bias toward a home‐range centroid. The direction of movement of individuals within a group was influenced by a group leader selected randomly at each time step with group members “following the leader” by constraining their movement direction to be within 30° of the direction directly toward the leader (see Gritter et al. 2024 for details).

**FIGURE 2:**
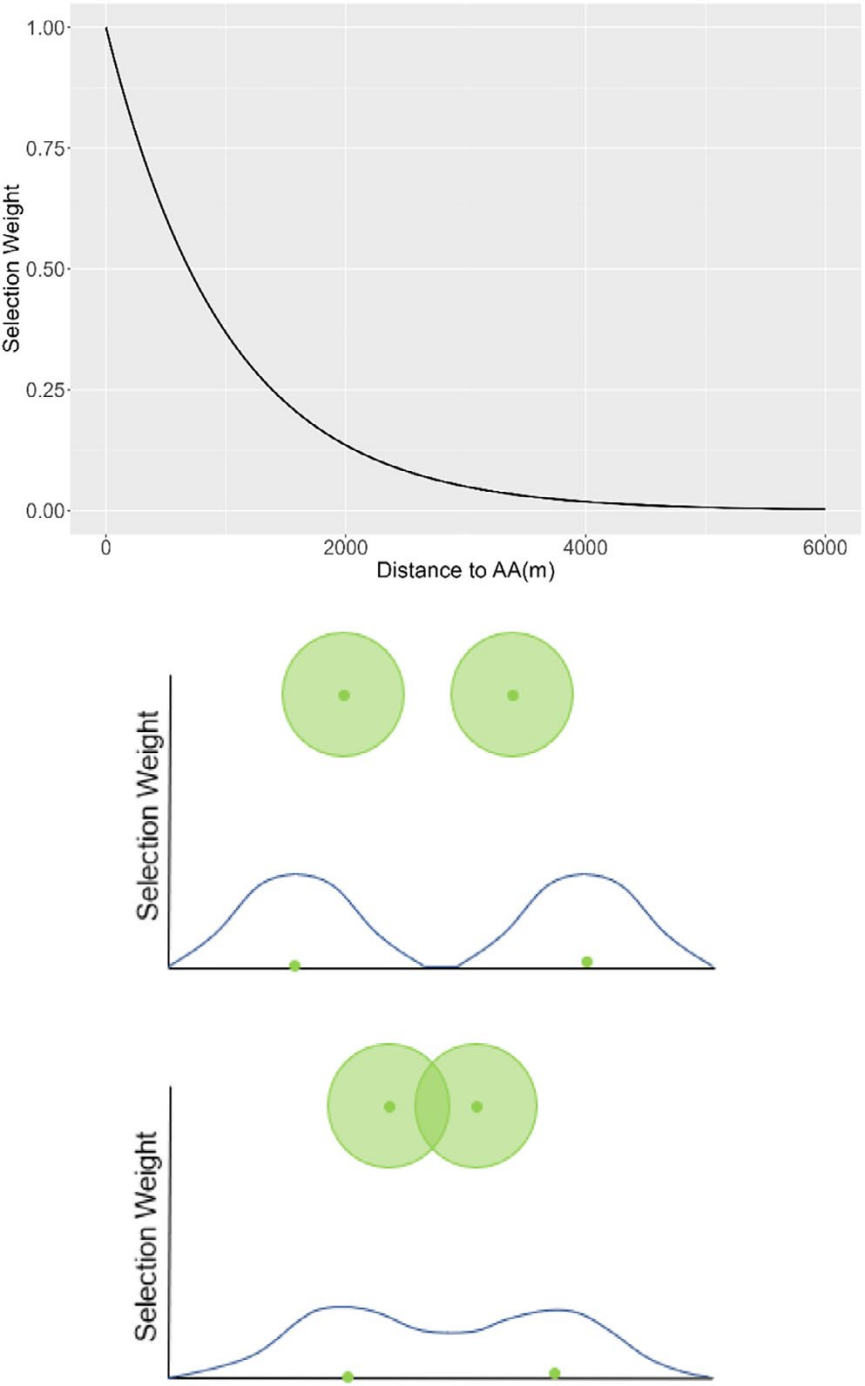
Top panel: Relationship between artificial‐attractant selection weight, in the step‐selection function for deer within the attraction range, and the distance to AA (Gritter et al. 2024). Bottom panels: Graphics illustrating the relative selection weights for areas around artificial attractants (green circles/dots) when they are distant (top) or near each other (bottom) as occurs in landscape with a low density and high density of artificial attractants, respectively.

Movement of a deer was biased toward AA when they came within a 6‐km buffer of the AA, by incorporating an attraction into the weighting function of the iSSFs with a negative beta coefficient of 0.001, producing a negative exponential relationship (Figure 2, top panel), so that weighting values were higher for cells (30 m by 30 m cells) closer to AA (McRae et al. 2020; smaller distance value). When any of the deer in a group landed on an AA or any of the 8 neighboring cells, attraction to AA for the entire group was eliminated for 24 h (12 timesteps) and the deer in the group moved only according to the non‐attractant landscape features included in the integrated step‐selection weighting function. We imposed this rule to prevent deer from staying around the site for the majority of the simulation; instead, deer moved away from the AA, back toward their home range.

### 2.3 Contact Simulations

We simulated deer movement and recorded contacts of deer in the same or different groups, the number of unique deer contacts, the number of unique AAs contacted by a deer, and the number of unique deer using an AA during a 144‐day winter period under 6 AA densities (0 AA to 1 AA/km^2^) and 6 AA configurations. The 6 spatial configurations represented two different agricultural practices of storing feed that potentially attract deer: grain bags and hay bales left within the fields where they were harvested (hereafter, field AA) and grain bins (where grain spillage can occur) and hay storage units, both of which typically are near farm sites (hereafter, farm AA). We focused on agricultural attractants and not supplemental salts/minerals, as previous studies have found deer use salt licks more in the spring/summer than in winter (Brochez et al. 2021; Schultz and Johnson 1992). Field AAs were randomly distributed within agricultural areas across the study area. Farm AAs were located based on buildings designated on the plat maps for Wainwright and Provost area (The Municipal District of Provost No. 52 2019; The Municipal District of Wainwright No. 61 2020), which assumed all buildings selected were farm sites (Figure 1). Placement of farm AAs was restricted to areas that were more than 400 m from the simulation area edge to ensure farm AAs were not placed outside the study area. Clusters of farm AAs were located around each farm centroid based on a bivariate normal spatial probability distribution, with a standard deviation of 200 m. We allocated farm AAs to farm sites used using a Neyman‐Scott process (Neyman and Scott 1952; Ripley 1977) that identified farm centroids as “parent” points and then placed the approximately equal number of “daughter” farm AAs around the parent.

Densities of AA were altered by reducing the maximum number of AA under a particular configuration for the field and farm AA scenarios. We altered densities of farm AA either by independently removing individual farm AA or by removing an entire cluster of farm AA at a farm (no AA remaining at the farm). We reduced AA randomly and by proximity to woody cover, where woody cover provides security cover/other resources for deer. The probability of removing AA based on distance to woody cover followed an exponential decay (coefficient = −0.0384) that was derived from the distribution of distances to woody cover values of GPS locations pooled across 25 male and 52 female from 16 December to 9 May (unpublished data). A cluster of AA was removed based on the mean distance to woody cover of all AA in the cluster.

We ran 5 iterations of deer moving across the landscape for each AA density × AA configuration. For all simulations, we kept the density of deer constant at 1/km^2^, which reflects the target density for disease containment (Bollinger et al. 2004). We defined a contact as two deer coming within 5 m of each other and classified a contact by whether it occurred between deer within the same (within) or in different (between) groups.

### 2.4 Sensitivity Analyses

We conducted a sensitivity analysis on the attraction range at which deer detect AA and the deer's strength of attraction to an AA to determine the robustness of our results because of a lack of data. Other model parameters have already been assessed (see Gritter et al. 2024). We expected that as the attraction range increased, deer would move between more attractants, leading them to visit more different AAs, which would be reflected in an increase in the number of AAs used and the number of deer using each site. In contrast, as the attraction to an AA increased (i.e., attraction beta increased) deer would be less likely to move among attractants and spend more time around fewer AAs, which would be reflected in decreasing numbers of AAs used and deer using each site. We varied the attraction range from 3000 to 9000 m with a step of 600 m, attraction beta from 0.0005 to 0.0015 with a step of 0.0001, and ran simulations at high (1 AA/km^2^) and low (0.2 AA/km^2^) densities. We conducted the sensitivity analysis using Latin hypercube sampling in the nlrx package in R and report the partial correlation coefficient, which indicates the strength of the linear relationship, and the partial rank correlation coefficient, which evaluates nonlinear relationships (Salecker 2019).

## 3 Results

**FIGURE 3:**
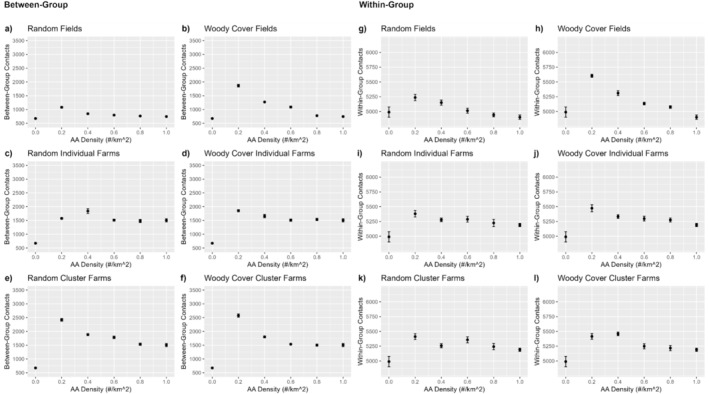
Mean (± SE) number of total contacts between‐groups (left) and within‐groups (right) averaged across 5 random‐seed iterations for each attractant (AA) density. See text for explanation of placement and removal strategies. Note scales of the y‐axes differ among panels.

The mean number of total contacts/deer (hereafter, deer contact rates) was the same for each scenario when there were no AA on the landscape because identical conditions were used in all these simulations (Figure 3). Similarly, deer contact rates were the same within field AA and farm AA simulations at maximum AA densities (1 AA/km^2^) because with no removal having occurred, the distribution of AA was identical within farm AA and within field AA scenarios. As expected, deer contact rates within groups were 2–7 times higher than between‐group contact across all scenarios (Figure 3). Deer contact rates both within and between groups initially increased as AA were removed under most simulated conditions (Figure 3). Between but not within group contact rates increased relatively more when farm AA were removed by cluster than when farm AA were removed independently. When field AA near woody cover were selectively removed, deer contact rates at field AA increased relatively more than when field AA were removed randomly.

**FIGURE 4:**
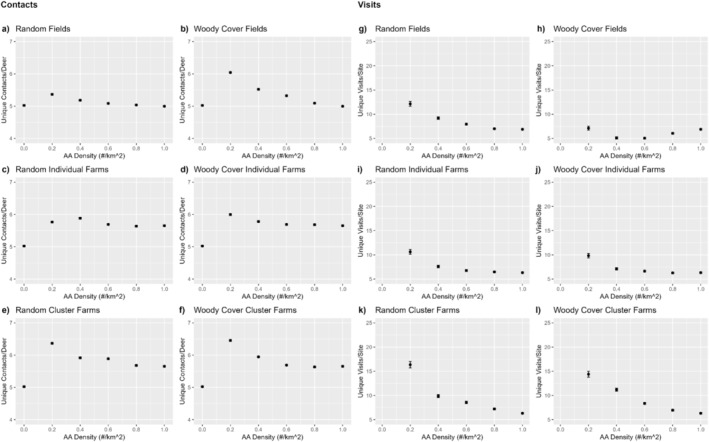
Mean (± SE) number of unique contacts with other deer (a–f) and number of unique deer that visited each attractant (g–l) averaged across 5 random‐seed iterations by AA density and AA placement and removal strategies. See text for explanation of placement and removal strategies.

Mean number of unique deer contacted by each individual deer was higher when AA were present on the landscape than when absent (Figure 4). Deer generally had contacts with more unique deer at low, rather than high AA densities, with deer contacting a higher number of unique deer when AA were removed near woody cover than at random. The highest number of unique deer contacted occurred when farm AA clusters were removed. Overall, the number of contacts across all scenarios varied only from 5.0 to 6.8 deer across all AA placement and removal strategies.

**FIGURE 5:**
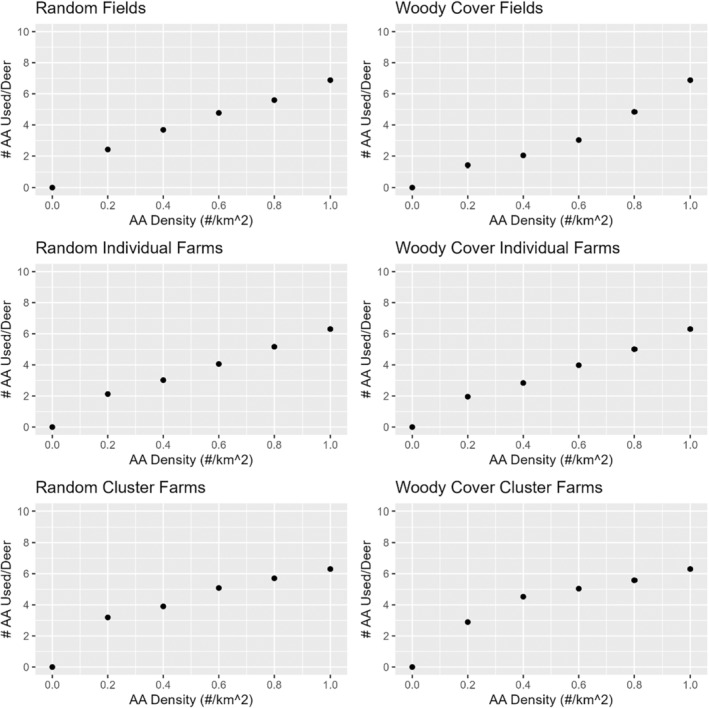
Mean (± SE) number of different artificial attractants (AA) used by each deer for 5 random‐seed iterations by AA density and AA placement and removal strategies. See text for descriptions of placement and removal strategies.

The number of different AA used by each deer increased almost linearly with AA density across scenarios, with some visual evidence that the relationship might have begun to saturate at high densities when AA were clustered around farms (Figure 5). In contrast, the number of unique deer visiting a particular AA was highest at low AA density across scenarios, with the highest number of unique deer visiting a particular AA at low density occurring when farm AA clusters were removed (Figure 4g–l). The removal of AA near woody cover generally resulted in fewer different deer visiting the AA than when removed randomly, with the effect most pronounced for field AA.

**FIGURE 6:**
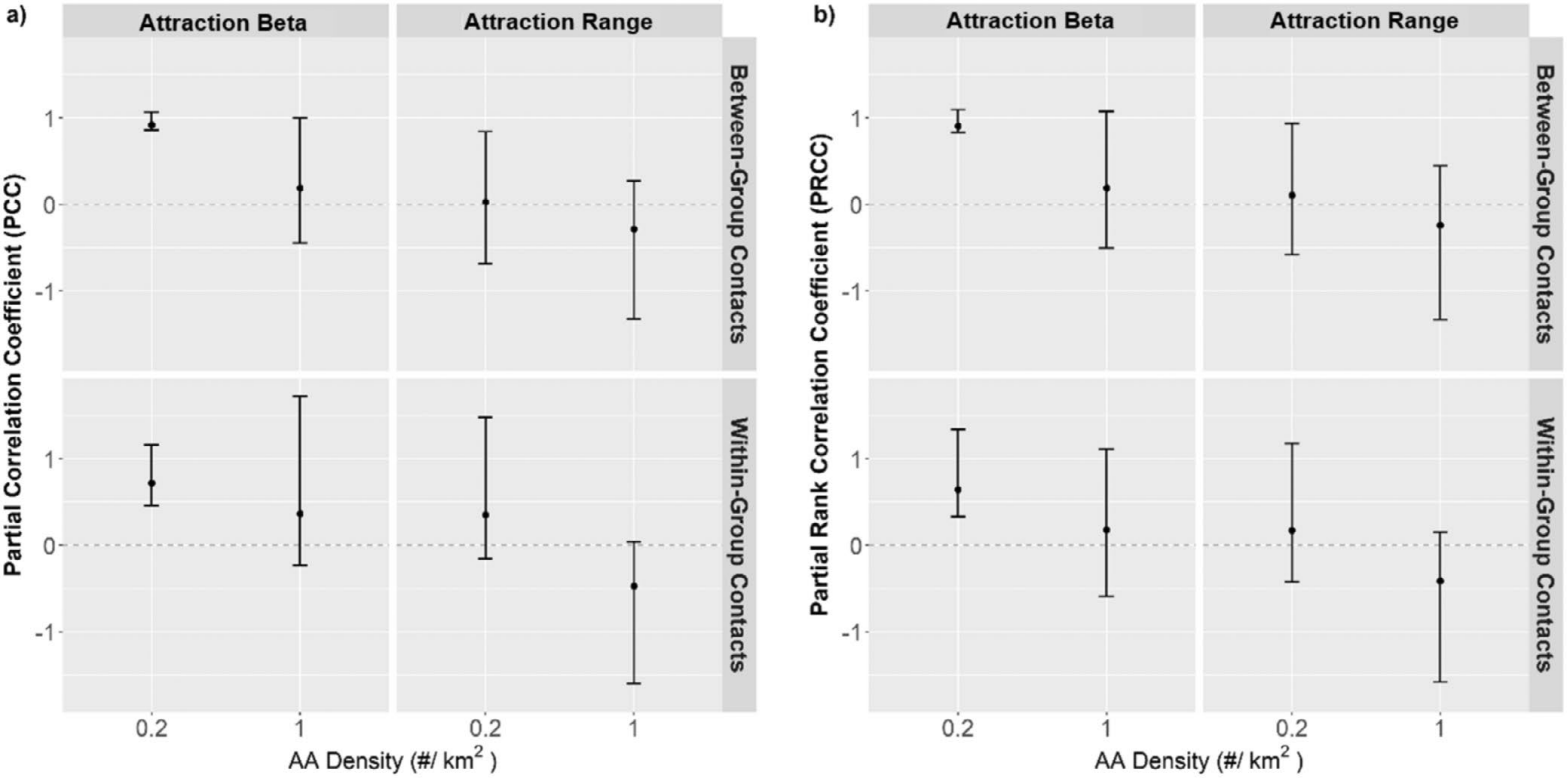
Sensitivity in within‐ and between‐group contact rates at a high (1 AA/km^2^) and low (0.2 AA/km^2^) density to the variation in beta coefficients (0.0005–0.0015), reflecting strength of attraction to AA, and to variation in the attraction range (3000–6000 m) at which deer detect and are assumed to be attracted to an AA. Presented are partial correlation coefficients (a), which indicate the strength of linear relationship, and the partial rank correlation coefficients (b), which evaluate for nonlinear relationships. Positive sensitivity values indicate that contact rates increase whereas negative values indicate contact rates decrease with an increase in variable value. Confidence intervals were obtained via bootstrapping.

Contact rates when AA were at their maximum density were more sensitive to the attraction range at which an AA was detected than to the strength of attraction once detected, but this was only marginally true as all confidence intervals overlapped zero (Figure 6). The negative correlation of ~0.25–0.5 indicates decreasing levels of contacts at increased attraction range (Figure 6). At a low density of AA, there was a greater effect of strength of attraction to AA on contact rates, with a correlation of ~0.75–1, indicating an increase in contacts with increasing attraction (Figure 6). The influence of attraction range also changed at low AA density, with the correlation approaching zero for between‐group contacts and becoming a positive relationship for within‐group contacts.

## 4 Discussion

Deer use of artificial attractants may be important in disease transmission, making guidelines for managing AA a key component of disease management (Sorensen 2014; Thompson et al. 2008). The most commonly proposed approach to managing agriculturally related AA, such as hay bales or bags and grain spillages, is to reduce or eliminate the number on the landscape (as in Mejía‐Salazar et al. 2018). The Western Association of Fish and Wildlife Agencies (Western Association of Fish and Wildlife Agencies 2018) recommends empirically evaluating a 65% reduction in AA in agricultural lands to limit the spread of CWD, which corresponds to the reduction we examined in this simulation study where we started with 1 AA/km^2^. Our simulations suggest outcomes of reducing AA may not be as intuitive as expected.

We found a nonlinear relationship between AA density and both within‐ and between‐group contact rates that showed the highest contact rates occurring at low AA/km^2^, declining as AA density increased. When we reduced the number of AA, the strong attraction to the remaining few isolated AA (i.e., high relative iSSF weight) increased contact rates with other deer or deer groups (Miller et al. 2003). When AA density is low, selection values are more variable because there is less overlap in the areas of high attraction around AAs (Figure 2). As a result, proportionally less of the landscape was attractive to deer, whereas deer were strongly attracted to any AA within their perception zone (Figure 2). The high contact rates at low AA density are maintained despite the attraction being relaxed for 24 h because deer typically do not move outside the 6000‐m attraction range. When deer find isolated AA, they tend to stay there. In contrast, as the number of AA increased, selection values across the landscape became relatively more homogeneous due to increased overlap of the attraction zones of AA (Figure 2). With more AA options on the landscape, deer are attracted to more of the available AA and could be attracted to 2 or more different AA from some locations.

When prevalence is low to moderate, reducing the number of AAs may contribute to a reduced risk of prion presence at a site. Individual deer visited fewer different AAs, and thus there was a lower risk of visiting a site where disease might be present. In contrast, when prevalence is high, it is likely at least one infected deer will visit most AAs. This could be particularly important for diseases like CWD, where infectious prions shed in bodily fluids (Mathiason et al. 2009; Saunders et al. 2012) could be accumulated, creating an environmental reservoir for indirect disease transmission (Miller and Williams 2004). However, conversely, repeated use of a single or few sites by an infected deer could cause a greater buildup of prions than when the infected individual uses multiple sites, increasing the risk of contracting CWD from the environment. Additionally, with an infected deer present at each site when there are fewer AAs, there is an increased risk of direct or indirect transmission both within the mule deer population and interspecies transmission. Barrile et al. (2024) found that mule deer females in the terminal stages of CWD modified their movement behavior and habitat selection, which could lead to them staying closer to the consistent food source of an AA. Key to the relative role of environmental transmission is the frequency of use of AAs by infected individuals and either prion deterioration or reduced prion access through leaching (Kuznetsova et al. 2014; Miller et al. 2003). In simulations of direct and indirect transmission, prion survival alone accounted for 29% and 31% of the variation in projected outcomes in CWD prevalence, and prevalence growth rate, peak prevalence, and R_0_ of a population were all expected to scale with the duration of prion persistence (Almberg et al. 2011). The accumulation of prions in the environment may continue to expose deer and other cervid species to CWD for some period even if the source of the attraction is eliminated (Jacobson et al. 2010; Miller and Williams 2004). Determining how long deer continue to return to feeding sites after sources are removed could be a productive area of research (Western Association of Fish and Wildlife Agencies 2018).

From a disease‐management perspective, our outcomes indicate that reducing the density of AA may not decrease contact rates or environmental exposure, but may increase the opportunity for spreading a disease like CWD that transmits by animal‐to‐animal contact and through the environment. However, we modeled contact rates under a moderate deer density for this region, and we did not explore how changes in deer density influenced contact rates in these scenarios. We suspect that with very low deer densities, where deer groups are broadly dispersed across the landscape, contact rates may decrease under low AA density because fewer deer will be within the perception range of AA, meaning that possibly fewer deer will find an AA, and if they do, it is less likely that there will be multiple groups at that AA. Apparent prevalence of bovine tuberculosis (*Mycobacterium bovis*) in white‐tailed deer in Michigan declined when deer density was reduced (O'Brien et al. 2011). In that case, restrictions on baiting and feeding occurred at the same time such that the effect of population reduction vs. the bans could not be distinguished. Even when populations are reduced, deer tend to congregate in preferred habitats, making it challenging to achieve low densities at local scales. In our simulations, we initially located deer on the landscape based on a home‐range RSF, which concentrated them in certain areas (Gritter et al. 2024). There also is evidence that the relationship between deer population size and crop damage is weak (Hewison et al. 2001; Kilpatrick and Stober 2002). Further simulations of the interaction between deer and AA densities on contact rates are warranted and may support AA reduction where deer densities are low or simultaneously reduced.

We also explored different approaches to removing AA from the landscape. Removing entire clusters of AA around farms led to increased contacts between groups compared to removing individual farm AA. When all AA at a farm were removed, deer groups that had been using those AA moved to nearby farms with AA, which increased between‐group contacts. Individuals remained in the same group regardless of whether some or all of the AA were removed, so within‐group contacts did not increase. Thus, removing some clusters of AA was not effective in reducing the opportunity for disease transfer. Even if all AA at a farm were eliminated, the management effort may still be thwarted. Some farmers are likely more amenable to working with managers to remedy AA problems, especially where agencies implement cost‐sharing or lending programs to support the implementation of solutions such as fencing (Mark Heckbert, pers. comm.). However, if only some farms reduce their AA, the opportunity for displaced deer to move to farms that still have AA undermines the overall success of disease management. This situation occurred with black‐backed jackals ( *Canis mesomelas* ) in Namibia where, when feeding site density decreased, contacts between individuals at feeding sites initially increased, followed by a decrease at very low feeding site density (Borchering et al. 2017). In our study area, farms themselves were clustered, often along major access roads. Reducing the influence of AA on deer contacts through working with a block of nearby farms to remove AA may offer opportunities to improve success.

Targeting AA near specific vegetation types preferred by deer could be used for managing disease in an agricultural landscape. Directing the removal of AA adjacent to natural habitats that provide cover and protection may be an incentive for deer to remain close to preferred habitat and not move to AA. We investigated the removal of AA near patches of woody cover both in fields and at farms because woodlands are preferred habitat in this area (Habib et al. 2011; Nobert et al. 2016). However, the removal of AA near these sites actually increased contacts between groups both when single AA in the fields or a full cluster of AA around a farm were removed. As in the situation of removing individual clusters of AA, deer simply moved to nearby AA, particularly those near woody cover. However, in our model, AA had a stronger attraction than preferred habitat per se. Although we had empirical data on the selection of environmental features (e.g., woody cover), we did not have empirical data on the strength of the attraction to AA to incorporate into our iSSF. A strong attraction to AA seems reasonable in winter as deer focus on AA as natural feed is less available (Mysterud et al. 2021). But studies that quantify the attraction to AA relative to the extent of natural habitat under a range of environments and costs of movements are needed to quantify ranges and strength of attraction to AA, particularly in light of simulation results indicating that when AA are eliminated, deer are likely to move to adjacent AA when sufficiently close. For example, investigating if AA are more attractive/problematic in severe winters or with high snow depth compared to milder winters is warranted; however, we suspect that the high forage value of grain spills and hay bales makes these food sources attractive in most winters.

Several other assumptions of how deer move and their attraction influenced our results and require further exploration in empirical settings. The strength of deer attraction to an AA once perceived had a greater influence on the number of contacts than the distance at which an AA was perceived, particularly at low AA density. We kept the attraction value and attraction range constant among deer groups, but in reality, these can differ. For example, the strength of attraction to AA may be highly variable depending on the type of attractant. Cosgrove et al. (2018) used variable attraction values to different kinds of feed piles in their individual‐based, spatially explicit model and found that attractiveness had a substantial effect on bovine tuberculosis prevalence. We also did not include group fission–fusion dynamics or limit the number of deer groups feeding at a site (Gritter et al. 2024). Although mule deer groups in winter tend to be relatively stable (Lingle 2001), low group cohesion can strongly influence contact rates (Aureli et al. 2012; Body et al. 2015) and would be expected to alter the number of unique deer visiting a site. Additionally, dominance and antagonism among individuals increase at artificial feeding sites (Peterson 2005; Thompson et al. 2008) and may restrict the time deer remain at an AA. Direct, systematic counts or measures of time spent at an AA would be useful in setting these constraints. Lastly, we did not account for the potential influence of memory in resource selection, which has been found to be a factor in a number of cervid species (Ranc et al. 2021; Kashetsky et al. 2021; Merkle et al. 2019) and could be a model development to investigate if this changes our conclusions.

Overall, deer responses to the removal of AA may not be straightforward, and further modeling and ultimately field experiments will be necessary to fully appreciate the role of AA in promoting disease spread. Our model outputs suggest four key aspects in designing these efforts: (1) the interaction between deer density and AA density for exposure to AA, (2) the strength of attraction of artificial feed sites relative to habitat conditions, (3) attraction range and how much time deer spend at AA, and (4) social and economic values of farmers that influence their willingness to address deer feeding on their lands. The latter is particularly important (Carstensen et al. 2011). Even if the best strategy were to regulate AA strongly, issues with compliance are likely to arise (Cosgrove et al. 2018; O'Brien et al. 2011). For example, in Norway, a ban aimed at removing all supplemental feeding sites was not successful, as it only reduced the percentage of municipalities feeding by < 12% (Mysterud et al. 2019). In the case of CWD, continued efforts to work with landowners to eliminate or reduce access to feeding sites, or to find alternative strategies such as rotating locations of grain bags over time, will be key to minimizing environmental hotspots of diseases that may persist even after sources of food are removed.

## Author Contributions

**Evelyn Merrill:** conceptualization (equal), data curation (lead), funding acquisition (equal), methodology (equal), supervision (equal), writing – review and editing (equal). **Mark A. Lewis:** conceptualization (equal), formal analysis (equal), funding acquisition (equal), methodology (equal), supervision (equal), writing – review and editing (equal). **Margo Pybus:** conceptualization (equal), data curation (supporting), methodology (equal), supervision (supporting), writing – review and editing (equal). **Kelsey Gritter:** conceptualization (equal), formal analysis (lead), methodology (equal), writing – original draft (lead), writing – review and editing (equal).

## Conflicts of Interest

The authors declare no conflicts of interest.

## Data Availability

All data are already presented in Gritter et al. (2024). IBM code and all data that are not publicly available elsewhere can be found at https://github.com/kgritter/IBM‐for‐Mule‐Deer. Data on wildlife damage complaint locations contain confidential information and cannot be shared.

## References

[ece371013-bib-0001] Almberg, E. S. , P. C. Cross , C. J. Johnson , D. M. Heisey , and B. J. Richards . 2011. “Modeling Routes of Chronic Wasting Disease Transmission: Environmental Prion Persistence Promotes Deer Population Decline and Extinction.” PLoS One 6: e19896. 21603638 10.1371/journal.pone.0019896 PMC3094393

[ece371013-bib-0002] Andreassen, H. P. , H. Gundersen , and T. Storaas . 2005. “The Effect of Scent‐Marking, Forest Clearing, and Supplemental Feeding on Moose‐Train Collisions.” Journal of Wildlife Management 69: 1125–1132.

[ece371013-bib-0068] Altalis . 2018, April 20. “25m Raster DEM.” Altalis. https://www.altalis.com/map.

[ece371013-bib-0069] Altalis . 2020, April 15. “Access.” Altalis. https://www.altalis.com/map.

[ece371013-bib-0003] Aureli, F. , C. M. Schaffner , N. Asensio , and D. Lusseau . 2012. “What Is a Subgroup? How Socioecological Factors Influence Interindividual Distance.” Behavioral Ecology 23: 1308–1315.

[ece371013-bib-0004] Austin, D. D. , P. J. Urness , and D. Duersch . 1998. “Alfalfa Hay Crop Loss due to Mule Deer Depredation.” Journal of Range Management 51: 29.

[ece371013-bib-0005] Barrile, G. M. , P. C. Cross , C. Stewart , et al. 2024. “Chronic Wasting Disease Alters the Movement Behavior and Habitat Use of Mule Deer During Clinical Stages of Infection.” Ecology and Evolution 14: e11418. 38779534 10.1002/ece3.11418 PMC11108800

[ece371013-bib-0006] Becker, D. , C. E. Snedden , S. Altizer , and R. J. Hall . 2018. “Host Dispersal Responses to Resource Supplementation Determine Pathogen Spread in Wildlife Metapopulations.” American Naturalist 192: 503–517. 10.1086/699477 30205031

[ece371013-bib-0007] Benavides, J. , P. D. Walsh , L. A. Meyers , M. Raymond , and D. Caillaud . 2012. “Transmission of Infectious Diseases en Route to Habitat Hotspots.” PLoS One 7: 31290. 10.1371/journal.pone.0031290 PMC3282722 22363606

[ece371013-bib-0008] Body, G. , R. B. Weladji , Ø. Holand , and M. Nieminen . 2015. “Fission‐Fusion Group Dynamics in Reindeer Reveal an Increase of Cohesiveness at the Beginning of the Peak Rut.” Acta Ethologica 18: 101–110.

[ece371013-bib-0009] Bollinger, T. , P. Caley , E. Merrill , et al. 2004. “Chronic Wasting Disease in Canadian Wildlife: An Expert Opinion on the Epidemiology and Risks to Wild Deer.” Canadian Cooperative Wildlife Health Centre.

[ece371013-bib-0010] Borchering, R. K. , S. E. Bellan , J. M. Flynn , J. R. C. Pulliam , and S. A. McKinley . 2017. “Resource‐Driven Encounters Among Consumers and Implications for the Spread of Infectious Disease.” Journal of the Royal Society, Interface 14: 20170555. 29021163 10.1098/rsif.2017.0555 PMC5665835

[ece371013-bib-0011] Brochez, C. B. , R. V. Rea , S. M. Crowley , and D. P. Hodder . 2021. “Year‐Round Patterns of Mineral Lick Use by Moose (*Alces americanus*), deer, and Elk (*Cervus canadensis*) in North‐Central British Columbia.” Canadian Field‐Naturalist 134: 361–374.

[ece371013-bib-0012] Carstensen, M. , D. J. O'Brien , and S. M. Schmitt . 2011. “Public Acceptance as a Determinant of Management Strategies for Bovine Tuberculosis in Free‐Ranging US Wildlife.” Veterinary Microbiology 151: 200–204. 21439739 10.1016/j.vetmic.2011.02.046

[ece371013-bib-0013] Conner, M. M. , M. E. Wood , A. Hubbs , et al. 2021. “The Relationship Between Harvest Management and Chronic Wasting Disease Prevalence Trends in Western Mule Deer (*Odocoileus hemionus*) Herds.” Journal of Wildlife Diseases 57: 831–843. 34648639 10.7589/JWD-D-20-00226

[ece371013-bib-0014] Cosgrove, M. K. , D. J. O'Brien , and D. S. L. Ramsey . 2018. “Baiting and Feeding Revisited: Modeling Factors Influencing Transmission of Tuberculosis Among Deer and to Cattle.” Frontiers in Veterinary Science 5: 306. 30564585 10.3389/fvets.2018.00306 PMC6288431

[ece371013-bib-0015] Cotterill, G. G. , P. C. Cross , E. K. Cole , et al. 2018. “Winter Feeding of Elk in the Greater Yellowstone Ecosystem and Its Effects on Disease Dynamics.” Philosophical Transactions of the Royal Society, B: Biological Sciences 373: 20170093. 10.1098/rstb.2017.0093 PMC5882999 29531148

[ece371013-bib-0016] Cross, P. C. , T. G. Creech , M. R. Ebinger , et al. 2013. “Female Elk Contacts Are Neither Frequency nor Density Dependent.” Ecology 94: 2076–2086. 24279278 10.1890/12-2086.1

[ece371013-bib-0017] Dobbin, M. A. , P. Smolko , L. Put , and E. H. Merrill . 2023. “Risky Business: Relating Probability of Direct Contact to Risk of Chronic Wasting Disease.” Frontiers in Ecology and Evolution 11: 1156853.

[ece371013-bib-0018] Escobar, L. E. , S. Pritzkow , S. N. Winter , et al. 2020. “The Ecology of Chronic Wasting Disease in Wildlife.” Biological Reviews 95: 393–408. 31750623 10.1111/brv.12568 PMC7085120

[ece371013-bib-0019] Felton, A. M. , A. Felton , J. P. G. M. Cromsigt , L. Edenius , J. Malmsten , and H. K. Wam . 2017. “Interactions Between Ungulates, Forests, and Supplementary Feeding: The Role of Nutritional Balancing in Determining Outcomes.” Mammal Research 62: 1–7.

[ece371013-bib-0020] Gooding, R. M. , and R. K. Brook . 2014. “Modeling and Mitigating Winter Hay Bale Damage by Elk in a Low Prevalence Bovine Tuberculosis Endemic Zone.” Preventive Veterinary Medicine 114: 123–131. 24486094 10.1016/j.prevetmed.2014.01.005

[ece371013-bib-0021] Government of Alberta . 2021. Wildlife Damage Complaints. Government of Alberta.

[ece371013-bib-0022] Gritter, K. , M. Dobbin , E. Merrill , and M. Lewis . 2024. “An Individual‐Based Movement Model for Contacts Between Mule Deer (*Odocoileus hemionus*).” Ecological Complexity 58: 101082.

[ece371013-bib-0023] Habib, T. J. , E. H. Merrill , M. J. Pybus , and D. W. Coltman . 2011. “Modelling Landscape Effects on Density–Contact Rate Relationships of Deer in Eastern Alberta: Implications for Chronic Wasting Disease.” Ecological Modelling 222: 2722–2732.

[ece371013-bib-0024] Heberlein, T. A. 2004. “‘Fire in the Sistine Chapel’: How Wisconsin Responded to Chronic Wasting Disease.” Human Dimensions of Wildlife 9: 165–179.

[ece371013-bib-0025] Hewison, A. J. , J. P. Vincent , J. Joachim , J. M. Angibault , B. Cargnelutti , and C. Cibien . 2001. “The Effects of Woodland Fragmentation and Human Activity on Roe Deer Distribution in Agricultural Landscapes.” Canadian Journal of Zoology 79: 679–689.

[ece371013-bib-0026] Jacobson, K. H. , S. Lee , R. A. Somerville , D. McKenzie , C. H. Benson , and J. A. Pedersen . 2010. “Transport of the Pathogenic Prion Protein Through Soils.” Journal of Environmental Quality 39: 1145–1152. 20830901 10.2134/jeq2009.0137 PMC3073504

[ece371013-bib-0027] Jerina, K. 2012. “Roads and Supplemental Feeding Affect Home‐Range Size of Slovenian Red Deer More Than Natural Factors.” Journal of Mammalogy 93: 1139–1148.

[ece371013-bib-0028] Kashetsky, T. , T. Avgar , and R. Dukas . 2021. “The Cognitive Ecology of Animal Movement: Evidence From Birds and Mammals.” Frontiers in Ecology and Evolution 9: 724887.

[ece371013-bib-0029] Kilpatrick, H. J. , and W. A. Stober . 2002. “Effects of Temporary Bait Sites on Movements of Suburban White‐Tailed Deer.” Wildlife Society Bulletin 30: 760–766.

[ece371013-bib-0030] Kuznetsova, A. , D. McKenzie , P. Banser , T. Siddique , and J. M. Aiken . 2014. “Potential Role of Soil Properties in the Spread of CWD in Western Canada.” Prion 8: 92–99. 24618673 10.4161/pri.28467 PMC7030902

[ece371013-bib-0031] Lemieux, N. , B. K. Maynard , and W. A. Johnson . 2000. “A Regional Survey of Deer Damage Throughout Northeast Nurseries and Orchards.” Journal of Environmental Horticulture 18: 1–4.

[ece371013-bib-0032] Lingle, S. 2001. “Anti‐Predator Strategies and Grouping Patterns in White‐Tailed Deer and Mule Deer.” Ethology 107: 295–314.

[ece371013-bib-0033] Mathiason, C. K. , S. A. Hays , J. Powers , et al. 2009. “Infectious Prions in Pre‐Clinical Deer and Transmission of Chronic Wasting Disease Solely by Environmental Exposure.” PLoS One 4: 5916. 10.1371/journal.pone.0005916 PMC2691594 19529769

[ece371013-bib-0034] McRae, J. E. , P. E. Schlichting , N. P. Snow , et al. 2020. “Factors Affecting Bait Site Visitation: Area of Influence of Baits.” Wildlife Society Bulletin 44: 362–371.

[ece371013-bib-0035] Meijer, M. , and E. Karpuk . 1999. Dillberry Lake Provincial Park Biophysical Inventory. Government of Alberta.

[ece371013-bib-0036] Mejía‐Salazar, M. F. , C. L. Waldner , Y. T. Hwang , and T. K. Bollinger . 2018. “Use of Environmental Sites by Mule Deer: A Proxy for Relative Risk of Chronic Wasting Disease Exposure and Transmission.” Ecosphere 9: 2055.

[ece371013-bib-0037] Menichetti, L. , L. Touzot , K. Elofsson , R. Hyvönen , T. Kätterer , and P. Kjellander . 2019. “Interactions Between a Population of Fallow Deer (*Dama dama*), Humans and Crops in a Managed Composite Temperate Landscape in Southern Sweden: Conflict or Opportunity?” PLoS One 14: 215594. 10.1371/journal.pone.0215594 PMC6478287 31013322

[ece371013-bib-0038] Merkle, J. A. , H. Sawyer , K. L. Monteith , S. P. H. Dwinnell , G. L. Fralick , and M. J. Kauffman . 2019. “Spatial Memory Shapes Migration and Its Benefits: Evidence From a Large Herbivore.” Ecology Letters 22: 1797–1805. 31412429 10.1111/ele.13362

[ece371013-bib-0039] Miller, M. W. , and E. S. Williams . 2004. Chronic Wasting Disease of Cervids. Springer. 10.1007/978-3-662-08441-0_8 15148993

[ece371013-bib-0040] Miller, R. , J. B. Kaneene , S. D. Fitzgerald , and S. M. Schmitt . 2003. “Evaluation of the Influence of Supplemental Feeding of White‐Tailed Deer (*Odocoileus virginianus*) on the Prevalence of Bovine Tuberculosis in the Michigan Wild Deer Population.” Journal of Wildlife Diseases 39: 84–95. 12685071 10.7589/0090-3558-39.1.84

[ece371013-bib-0041] Milner, J. M. , F. M. Van Beest , K. T. Schmidt , R. K. Brook , and T. Storaas . 2014. “To Feed or Not to Feed? Evidence of the Intended and Unintended Effects of Feeding Wild Ungulates: Effects of Feeding Ungulates.” Journal of Wildlife Management 78: 1322–1334.

[ece371013-bib-0042] Mysterud, A. , and C. M. Rolandsen . 2019. “Fencing for Wildlife Disease Control.” Journal of Applied Ecology 56: 519–525.

[ece371013-bib-0043] Mysterud, A. , I. N. Skjelbostad , I. M. Rivrud , Ø. Brekkum , and E. L. Meisingset . 2021. “Spatial Clustering by Red Deer and Its Relevance for Management of Chronic Wasting Disease.” Animals 11: 1272. 33925184 10.3390/ani11051272 PMC8146590

[ece371013-bib-0044] Mysterud, A. , H. Viljugrein , E. J. Solberg , and C. M. Rolandsen . 2019. “Legal Regulation of Supplementary Cervid Feeding Facing Chronic Wasting Disease.” Journal of Wildlife Management 83: 1667–1675.

[ece371013-bib-0045] Neyman, J. , and E. L. Scott . 1952. “A Theory of the Spatial Distribution of Galaxies.” Astrophysical Journal 116: 144.

[ece371013-bib-0046] Nobert, B. R. , E. H. Merrill , M. J. Pybus , T. K. Bollinger , and Y. T. Hwang . 2016. “Landscape Connectivity Predicts Chronic Wasting Disease Risk in Canada.” Journal of Applied Ecology 53: 1450–1459.

[ece371013-bib-0047] O'Brien, D. J. , S. M. Schmitt , S. D. Fitzgerald , and D. E. Berry . 2011. “Management of Bovine Tuberculosis in Michigan Wildlife: Current Status and Near‐Term Prospects.” Veterinary Microbiology 151: 179–187. 21414734 10.1016/j.vetmic.2011.02.042

[ece371013-bib-0048] Peterson, C. 2005. Mule Deer and Emergency Winter‐Feeding (M.S.). Utah State University.

[ece371013-bib-0049] Peterson, M. J. , M. D. Samuel , V. F. Nettles , G. Wobeser , and W. D. Hueston . 2002. Review of Chronic Wasting Disease Management Policies and Programs in Colorado Report. Colorado Division of Wildlife.

[ece371013-bib-0050] Ranc, N. , P. R. Moorcroft , F. Ossi , and F. Cagnacci . 2021. “Experimental Evidence of Memory‐Based Foraging Decisions in a Large Wild Mammal.” Proceedings of the National Academy of Sciences of the United States of America 118: e2014856118. 33837149 10.1073/pnas.2014856118 PMC8053919

[ece371013-bib-0051] Ripley, B. D. 1977. “Modelling Spatial Patterns.” Journal of the Royal Statistical Society: Series B: Methodological 39: 172–192.

[ece371013-bib-0052] Rivera, N. A. , A. L. Brandt , J. E. Novakofski , and N. E. Mateus‐Pinilla . 2019. “Chronic Wasting Disease in Cervids: Prevalence, Impact and Management Strategies.” Veterinary Medicine 10: 123–139. 31632898 10.2147/VMRR.S197404 PMC6778748

[ece371013-bib-0053] Sah, P. , J. Mann , and S. Bansal . 2018. “Disease Implications of Animal Social Network Structure: A Synthesis Across Social Systems.” Journal of Animal Ecology 87: 546–558. 29247466 10.1111/1365-2656.12786

[ece371013-bib-0070] Salecker, J. , M. Sciaini , K. M. Meyer , and K. Wiegand . 2019. “The nlrx r Package: A Next‐Generation Framework for Reproducible NetLogo Model Analyses.” Methods in Ecololgy and Evolution 10: 1854–1863.

[ece371013-bib-0054] Saunders, S. E. , S. L. Bartelt‐Hunt , and J. C. Bartz . 2012. “Occurrence, Transmission, and Zoonotic Potential of Chronic Wasting Disease.” Emerging Infectious Diseases 18: 369–376. 22377159 10.3201/eid1803.110685 PMC3309570

[ece371013-bib-0055] Schultz, S. R. , and M. K. Johnson . 1992. “Effects of Supplemental Mineral Licks on White‐Tailed Deer.” Wildlife Society Bulletin (1973–2006) 20: 303–308.

[ece371013-bib-0056] Smolko, P. , D. Seidel , M. Pybus , A. Hubbs , M. Ball , and E. Merrill . 2021. “Spatio‐Temporal Changes in Chronic Wasting Disease Risk in Wild Deer During 14 Years of Surveillance in Alberta, Canada.” Preventive Veterinary Medicine 197: 105512. 34740023 10.1016/j.prevetmed.2021.105512

[ece371013-bib-0057] Sorensen, A. 2014. Habitat Selection by Sympatric Ungulates in an Agricultural Landscape: Implications for Disease Transmission and Human‐Wildlife Conflict. Master's thesis, University of Saskatchewan.

[ece371013-bib-0058] Sorensen, A. A. , F. M. Beest , and R. K. Brook . 2015. “Quantifying Overlap in Crop Selection Patterns Among Three Sympatric Ungulates in an Agricultural Landscape.” Basic and Applied Ecology 16: 601–609.

[ece371013-bib-0059] The Municipal District of Provost No. 52 . 2019. “Ownership Map M.D. of Provost No. 52”. https://www.mdprovost.ca/.

[ece371013-bib-0060] The Municipal District of Wainwright No. 61 . 2020. “Ownership Map M.D. of Wainwright No. 61”. https://www.mdwainwright.ca/welcome‐to‐the‐m‐d‐of‐wainwright‐no‐61/ownership‐map/.

[ece371013-bib-0061] Thompson, A. , M. Samuel , and T. V. Deelen . 2008. “Alternative Feeding Strategies and Potential Disease Transmission in Wisconsin White‐Tailed Deer.” Journal of Wildlife Management 72: 416–421.

[ece371013-bib-0062] U.S. Geological Survey . 2024. “Distribution of Chronic Wasting Disease in North America”. https://www.usgs.gov/media/images/distribution‐chronic‐wasting‐disease‐north‐america‐0.

[ece371013-bib-0063] Western Association of Fish and Wildlife Agencies . 2018. Recommendations for Adaptive Management of Chronic Wasting Disease in the West. Western Association of Fish and Wildlife Agencies.

[ece371013-bib-0064] White, L. A. , J. D. Forester , and M. E. Craft . 2018. “Disease Outbreak Thresholds Emerge From Interactions Between Movement Behavior, Landscape Structure, and Epidemiology.” Proceedings of the National Academy of Sciences of the United States of America 115: 7374–7379. 29941567 10.1073/pnas.1801383115 PMC6048514

[ece371013-bib-0065] Wilensky, U. 1999. NetLogo. Center for Connected Learning and Computer‐Based Modeling, Northwestern University. http://ccl.northwestern.edu/netlogo/.

[ece371013-bib-0066] Williams, E. , and M. Miller . 2003. “Prion Disease Horizontal Prion Transmission in Mule Deer.” Nature 425: 35–36. 12955129 10.1038/425035a

[ece371013-bib-0067] Wywialowski, A. P. 1994. “Agricultural Producers' Perceptions of Wildlife‐Caused Losses.” Wildlife Society Bulletin 22: 370–382.

